# Vaso-Renal Change Versus Bright’s Disease

**Published:** 1887-10

**Authors:** 


VASO-RENAL CHANGE VERSUS BRIGHT'S DISEASE. By J. MILNER FOTHERGILL, M. D., EDIN. Pp. xii. and 219. NEW YORK: G. P. PUTNAM'S SONS. 1887. CHICAGO: W. T. KEENER.

This volume will be a welcome addition to the list of the author's works, to all those who like his writings, either because of or despite, their peculiar literary style, or because of their suggestiveness and practical value. He deprecates his own unfitness for the task, which his title implies, and says that he has attempted it because it ought be performed by someone, and there seems to be no other volunteer. He claims that the high vascular tension characteristic of the class of diseases under discussion is due to irritation caused by the presence of uric acid on the blood, and that all the subsequent tissue changes are accounted for by this high tension. The author is more of a medical philosopher than a scientific investigator, and admits that most of his pathological histology and experimental physiology is derived from the work of others, but the development of his thesis is in his own peculiar and discursive style. The text is very fully illustrated with lithographic plates drawn from actual microscopical preparations, and this part of the work has been done with an accuracy, skill and care which leave nothing to be desired in the drawings as they appear in the book. The printing and binding are also admirable.

